# Preparation of Micro-Pit-Textured PCD Tools and Micro-Turning Experiment on SiCp/Al Composites

**DOI:** 10.3390/mi13071141

**Published:** 2022-07-19

**Authors:** Xu Wang, Valentin L. Popov, Zhanjiang Yu, Yiquan Li, Jinkai Xu, Qiang Li, Huadong Yu

**Affiliations:** 1 National and Local Joint Engineering Laboratory for Precision Manufacturing and Detection Technology, Changchun University of Science and Technology, Changchun 130012, China; wangxu0119@163.com (X.W.); liyiquan@cust.edu.cn (Y.L.); xujinkai2000@163.com (J.X.); 2 Ministry of Education Key Laboratory for Cross-Scale Micro and Nano Manufacturing, Changchun University of Science and Technology, Changchun 130012, China; 3 Technische Universität Berlin, Department of System Dynamics and Friction Physics, 10623 Berlin, Germany; qiang.li@tu-berlin.de; 4 School of Mechanical and Aerospace Engineering, Jilin University, Changchun 130015, China; yuhuadong@cust.edu.cn

**Keywords:** SiCp/Al composites, PCD tool, texture, micro-pits with rounded corner, micro-turning, secondary cutting

## Abstract

Serious tool wear occurs very often during machining due to the reinforcing phases in the workpiece. In this study, micro-pit-textures were prepared on the surfaces of PCD tools with a nanosecond laser to improve their cutting performance on SiCp/Al composites. The micro-pits were designed with rounded corners to improve the chip flow. The location and size of the texture were determined by analyzing the tool-chip contact area of the non-textured tool. The cutting performance of these textured PCD tools was investigated through orthogonal cutting experiments. It was found that the optimal cutting performance of the textured tools was achieved with the proper distance of the texture from the main cutting edge (35 μm) and the pit spacing (60 μm), aa a result of which the main cutting force reduced by about 14%, and the tool wear and surface adhesion significantly reduced. This texture was then applied in the micro-turning experiments of the PCD tool on the SiCp/Al composites. The cutting force in this experiment reduced by 22%, and the textured tool provided better chip transfer and tool anti-tipping. In this study, the role of SiC particles as a third body between the tool and the chip surface is discussed.

## 1. Introduction

The SiCp/Al composite consists mainly of aluminum alloy as the matrix and SiC particles as the reinforcing phase. It has been widely used in many cutting-edge fields, such as aerospace, automobiles, electronics, and medical and optical instruments [1,2,3,4,5,6]. Due to the large elastoplasticity of the aluminum matrix and the high hardness of SiC particles, serious tool wear, adhesion, and tipping occur easily during the machining process. Much research has been conducted to improve the machining performance of this material [7,8,9]. PCD tools have high hardness and low friction coefficients, and are therefore widely used for machining SiCp/Al composites, carbon fiber composites, and many difficult-to-machine materials due to their good cutting stability. Soares et al. [10] compared the performance of PCD tools and cemented carbide tools for machining SiCp/Al composites, and the PCD tool surface was found to be more complete. Xu et al. [11] investigated the machinability of two different aluminum matrix composites machined by four cutting tools—a fine-grained cemented carbide tool, a ceramic tool, a CBN tool, and a PCD tool. It was found that the PCD tools had the greatest wear resistance and are suitable for machining hybrid reinforced aluminum-matrix composites. When machining fiber-reinforced the aluminum matrix composite, the fine-grained carbide tool performed well, with a low wear rate, good surface integrity, and the lowest machining cost. When cutting particle-reinforced aluminum matrix composites, PCD tools had better wear resistance, higher fracture resistance and lower adhesion than PCBN tools [12,13].

Many studies have shown that textured tools have great advantages in improving cutting performance [14,15]. The experiments of turning hardened-steel GCr15 materials by using a PCBN textured tool showed that a proper micro-pit texture can significantly reduce tool wear and improve the quality of the machined surface [16,17]. Su et al. [18] studied the performances of micro-textured PCD tools in cutting titanium alloy, including the cutting force, cutting temperature, and chip adhesion of the PCD micro-groove tool. The textured tools showed anti-friction and anti-adhesion effects. Song et al. [19] proposed a new method to actively control the lubrication state of the tool–chip interface in order to improve the tribological properties of the tool surface. The results showed that the textured tool effectively reduced the cutting force and cutting temperature, and that the tool’s anti-adhesion ability was improved. Xing et al. [20] prepared micro-grooves on PCD tool surfaces and investigated the influence of picosecond-laser-machining parameters (laser power, frequency, and scanning speed) on the surface quality, material removal rate, and surface roughness. The best machining performance was achieved through multi-objective optimization. The experiment by Zhang et al. [21] showed that when cutting AISI 1045 hardened steel by using carbide-based Ti55Al45N-coated tools, the textured tools effectively reduced the cutting force, tool adhesion, cutting temperature, and friction coefficient of the tool–chip interface. The influence of the texture shape and the addition of a solid lubricant in the texture on the cutting performance was studied in [22,23].

When cutting SiCp/Al composites by using PCD tools, the aluminum matrix causes serious tool adhesion and wear, and hard SiC particles generate high friction at the interface. Therefore, texturing the tool surface could be a good option. In this study, orthogonal cutting experiments were performed on SiCp/Al composites with textured PCD tools to study the influence of the micro-pit position and density on the cutting performance. The obtained optimal texture was then applied to the tools in turning experiments to demonstrate its practicability.

## 2. Materials and Methods

### 2.1. Experimental Equipment and Method

**Figure 1:**
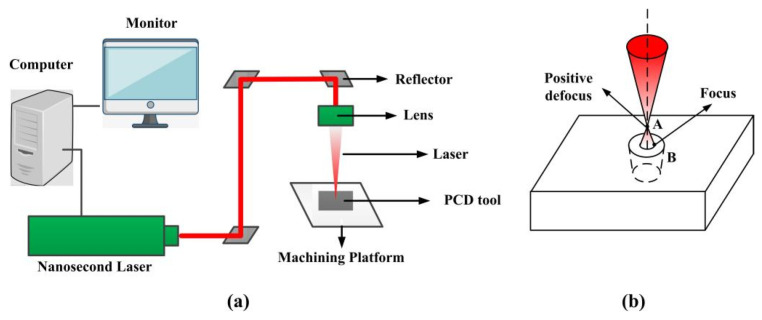
Schematic of (**a**) laser-control system and optical system, (**b**) schematic of laser machining.

A PCD tool with a medium grain (0.01 mm) was used (supplied by Xiamen Golden Egret Special Alloy Co. Ltd., Xiamen, China). Its density was 4.25 g/cm^3^, and the elasticity modulus and Poisson’s ratio were 1147 MPa and 0.07. Its hardness was approximately 8000 HV (3–5 times higher than that of cemented carbide) and the thermal conductivity was 700 W/(m K). The nanosecond laser was used to prepare textures on the PCD tool surface. The high laser power of 20 W was selected due to the high hardness of PCD tools. The laser-control system and optical system are shown in Figure 1a. Since the laser had an asymmetric active area, the focused spot was elliptical. The lens used to focus the laser beam onto the stage was an M Plan Apo NIR 50X (Mitutoyo Co., Kanagawa, Japan) with a NA of 0.42 and a focal length of 4 mm. The M^2^ size was less than 1.4. The laser wavelength was 1064 nm, and the pulse duration and frequency were 100 ns and 20 kHz, respectively. The outgoing beam diameter was 6.5 mm. The processing method of the texture tool was positive defocusing, as shown in Figure 1b; consequently, a micro-pit with a chamfer was prepared.

**Figure 2:**
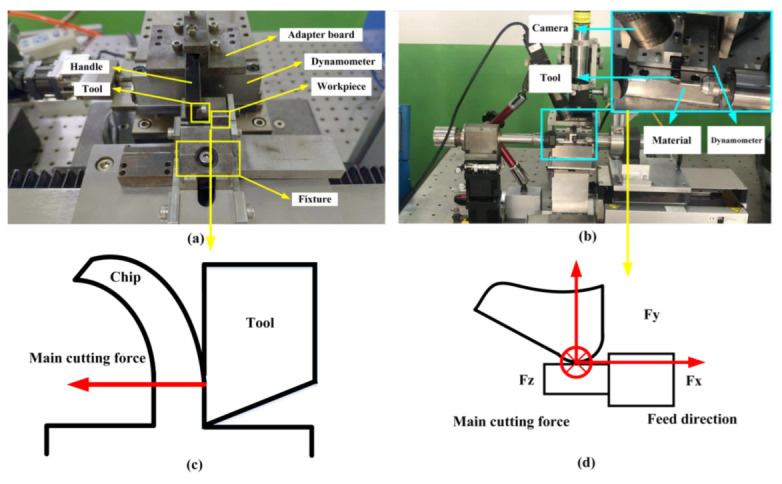
Experimental setups for orthogonal cutting (**a**) and micro-turning (**b**). Cutting forces are shown in (**c**,**d**).

The experimental setups for orthogonal cutting and micro-turning are shown in Figure 2. In the orthogonal cutting experiment, the workpiece made of SiCp/Al composite materials with sizes 10 × 10 × 0.8 mm was cut 20 times, and the cutting forces were averaged. The cutting depth was 0.02 mm and the cutting speed was 200 mm/s. In the turning experiment, a cylindrical bar with a diameter of 5 mm was selected. The cutting speed was 3000 rpm, the feed rate was 10 mm/min, the cutting depth was 0.015 mm, the turning length was 20 mm/time, and the cutting results were evaluated after 10 turnings. The definition of cutting forces is shown in Figure 2c,d.

### 2.2. Texture Design

**Figure 3:**
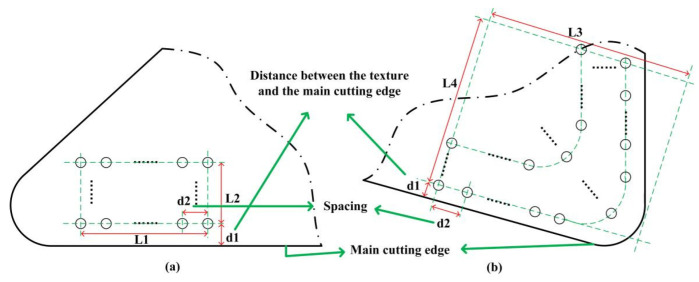
The design of micro-pit texture on PCD tool surface for (**a**) orthogonal cutting and (**b**) micro-turning experiments.

The location and size of textures were based on the orthogonal cutting experiment of non-textured tools in order to place the texture in complete contact with chip in the later machining experiment of textured tools. The tool–chip interaction area in case of non-textured tools was detected by SEM images. After the area observation, the texture size for orthogonal cutting experiment was designed as *L*_1_ × *L*_2_ = 200 μm × 1000 μm, as shown in Figure 3a, and the size for micro-turning experiment was *L*_3_ × *L*_4_ = 500 μm × 500 μm, as shown in Figure 3b. In orthogonal cutting experiments, the texture position was determined by the distance *d*_1_ between the first row of pits and the main cutting edge, and pit density was determined by the pit spacing *d*_2_. These two parameters varied as follows: *d*_1_—25 μm, 35 μm, and 45 μm; and *d*_2_—20 μm, 40 μm, 60 μm, 80 μm, and 100 μm. These results were combined in 15 textured tools in addition to one non-textured tool. The tools are enumerated in Table 1.

In the subsequent micro-turning experiments, only the non-textured tool and the textured tool that had the optimal cutting performance were selected for turning SiCp/Al composites.

**Figure 4:**
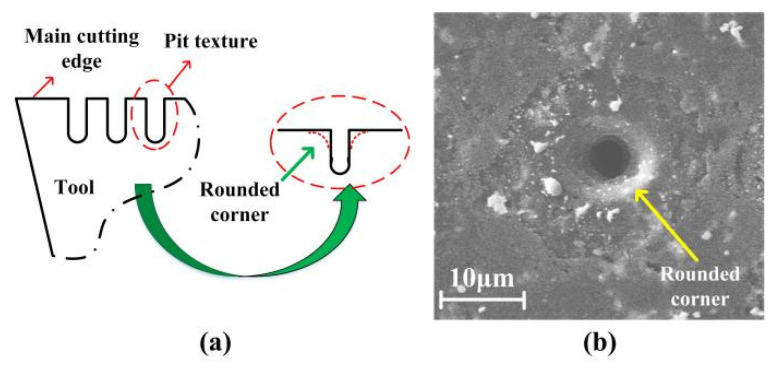
(**a**) Schematic diagram of micro-pits with rounded corners; (**b**) SEM image of one-micro pit before cutting.

It has been argued that the sharp corners of textures can increase the interaction of textures on chips, thereby producing serious secondary cutting [24,25]. Therefore, the micro-pits were prepared with rounded corners to avoid this effect and improve the machining performance, as shown in Figure 4. The pits were prepared with depth of approximately 30 µm, and inner and outer diameter of 5 and 10 µm. The image of the pits can be seen in the section of results. Note that the SiC particles in SiCp/Al composites had a size of 2 to 12 μm, in order of pit size.

**Figure 5:**
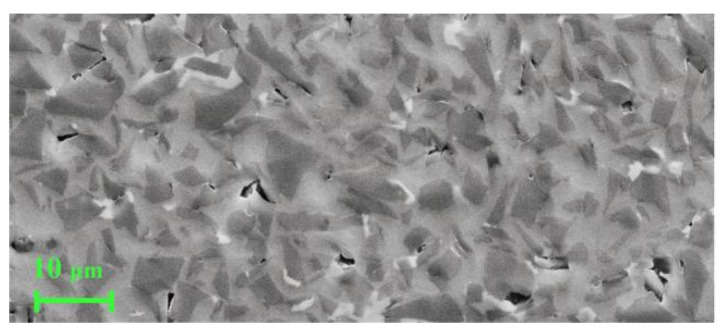
SEM image of SiCp/Al composite.

The properties of SiCp/Al composites provided by Harbin Institute of Technology are listed in Table 2. Note that the SiC particles in SiCp/Al composites had a size of 2 to 12 μm, in order of pit size. A SEM image of SiCp/Al composite is shown in Figure 5.

## 3. Results and Discussion

The influence of the texture parameters *d*_1_ and *d*_2_ on the orthogonal cutting and micro-turning performance is discussed in this section, including the cutting force, tool wear, adhesion, and chip form.

### 3.1. Tool Wear and Adhesion

**Figure 6:**
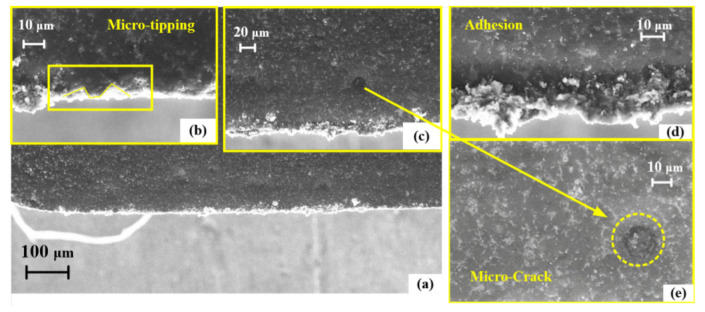
The SEM image of (**a**) non-textured tool surface after orthogonal cutting; (**b**)micro-tipping; (**c**) micro-crack; (**d**) adhesion; (**e**) micro-crack.

Figure 6 shows an SEM image of the non-textured tool surface after the orthogonal cutting of the SiCp/Al composite was performed 20 times. Since the SiCp/Al composite contained hard reinforcing SiC particles, the tool wear was very serious. It can be seen from Figure 6a,c,e that there were many micro-cracks with circular shapes, which were mainly generated by the pressing of the SiC particles onto the tool surface during machining. The size of the crack area was close to the particle size (2 to 12 μm). Due to the high plasticity of the aluminum matrix, serious adhesive wear occurred at the main cutting edge, as shown in Figure 6d. Meanwhile, since the SiC particles were in contact with the main cutting edge for a long time, micro-tipping can be observed, as shown in Figure 6b.

**Figure 7:**
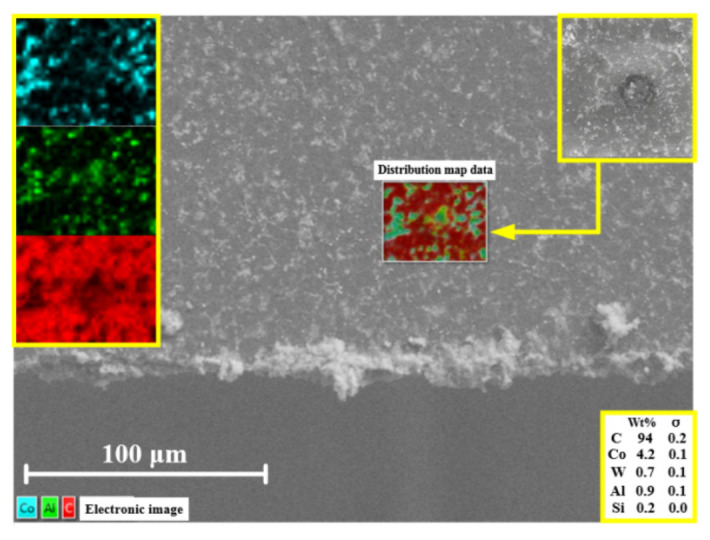
Energy-spectrum image of non-textured PCD tool near a crack.

Figure 7 shows the energy-spectrum image of the non-textured tool surface in the area near the crack (black spot). Carbon was mainly found to be distributed in this region, with only a small amount of aluminum and silicon. This was caused by the adhesive SiC particles indenting into the tool surface, as well as some broken SiC particles during machining. The aluminum matrix was pulled into the grooves as the chips slid on the tool surface.

**Figure 8:**
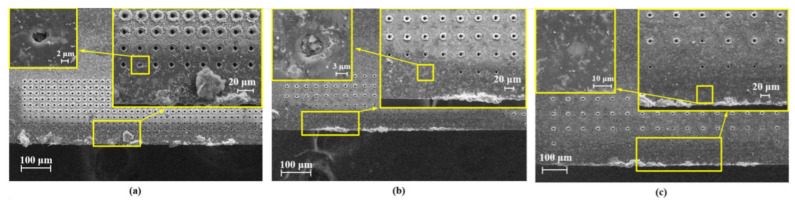

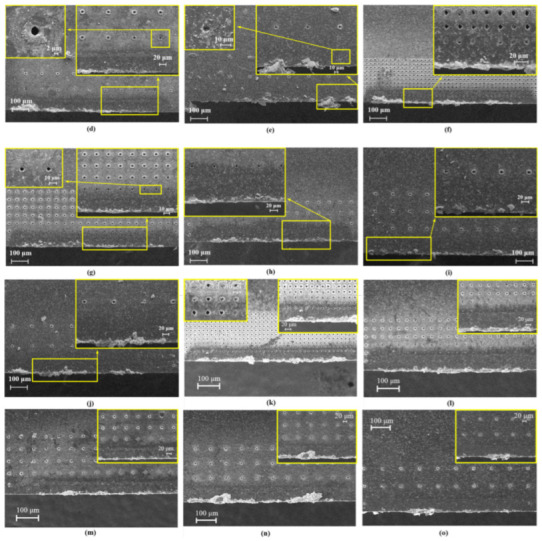
SEM images of micro-pit-textured PCD tool surface after orthogonal cutting. (**a**–**o**) Correspond to Tools 2 to 16.

The SEM images of surfaces of the 15 textured PCD tools after the cutting was performed 20 times are shown in Figure 8. The large-area adhesion, tool wear, and surface scratches were significantly reduced in these cases. Observing the tool adhesion, the textured tools with a smaller distance between the first row of pits and the main cutting edge (*d*_1_ = 25 μm, 35 μm) and relatively small pit spacing (*d*_2_ = 40 μm, 60 μm) exhibited better anti-adhesion (Figure 8b,c,g,h). When the texture spacing was too small (*d*_2_ = 20 μm) or too large (*d*_2_ = 80 μm and 100 μm), the anti-adhesion effect tended to decrease.

It was found that some micro-pits were blocked by residual materials, especially when they were close to the main cutting edge. By observing the locations of the blocked micro pits, it was possible to estimate the boundary of the sticking area. For example, in the case presented in Figure 8e (*d*_1_ = 25 μm and *d*_2_ = 60 μm), the first row of pits was completely blocked, but the second row was not. The same phenomenon can also be observed in Figure 8c (*d*_1_ = 25 μm and *d*_2_ = 20 μm). Comparing all the cases, the sticking area was estimated to be about 25–45 μm from the main cutting edge.

It was also noted that some of the pits were blocked by residual SiC particles rather than chips. These pits were mainly located in the sliding area. It was surmised that the SiC particles may play a supporting or rolling role between the chip and the texture during machining. The particle–chip interaction can increase the curvature of the chip and reduce the contact length of the chip, thereby reducing tool wear and adhesion.

### 3.2. Chip-Surface Analysis

**Figure 9:**
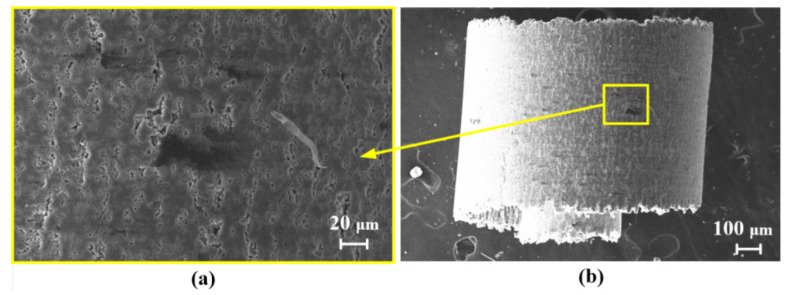
SEM image of chip surface machined by non-textured PCD tool. (**a**) Black trace on the chip surface; (**b)** SEM image of chip surface machined by non-textured PCD tool.

As in the previous section, the SEM image of the chip machined by the non-textured PCD tool is first shown in Figure 9. The whole chip was not broken, but many cracks were observed on the chip surface. Furthermore, some black traces appeared on the surface (Figure 9a). Its width was around 10 μm, which roughly corresponds to the size of the SiC particles. The reason for its formation may be that the local heat was difficult to dissipate due to the low thermal conductivity of the SiC particles, leading to an oxidation reaction on the surface of the chip, resulting in the abrasion of the particles. The dark and light black traces indicate that some of the particles rolled and other were scratched at the interface.

**Figure 10:**
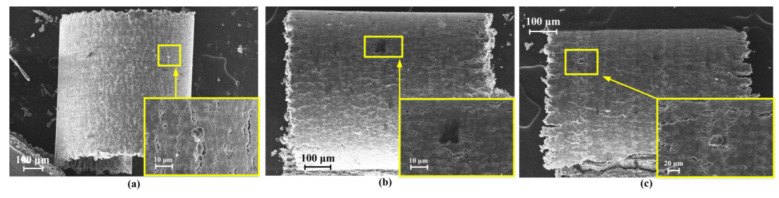

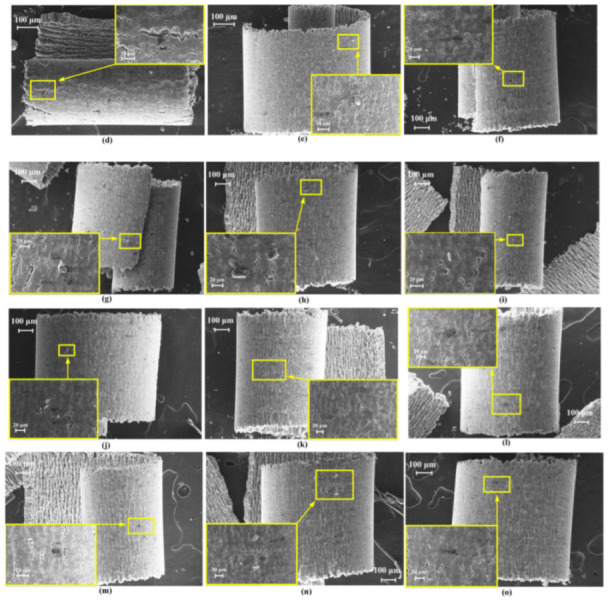
SEM images of chip surfaces machined by micro-pit-textured PCD Tools. (**a**–**o**) correspond to tools 2 to 16.

SEM images of the chips machined by the micro-pit textured PCD tool are shown in Figure 10. Compared to the case of the non-textured tool, these chips had larger curvature, and the chips appeared to break more easily. When the texture was far from the main cutting edge (increase of *d*_1_), the chip curvature obviously decreased (see, for example, the value of *d*_2_ = 60 μm in Figure 10f,h,m). As this distance increased, fewer micro-pits interacted with the chip; the chip curvature therefore tended to be that of a non-textured tool.

It is known that the micro-texture can cause secondary cutting. It can be affected by the pit shape and pit spacing. In this study, the pits were prepared with rounded corners. In the SEM images in Figure 10, it can be seen that in the case in Figure 10a, with *d*_1_ = 25 μm and *d*_2_ = 20 μm, there were slight scratches on the chip surface, which were produced by secondary cutting. In the other cases, secondary cutting was hardly observed. Compared with the sharp corners, the rounded corners on the micro-pits improved the smooth flow of the chips on the tool. In addition, the presence of some residual SiC particles on both the chip surface and the tool surface (Figure 8) indicated a three-body interaction between the tool surface, SiC particles, and chips. Some SiC particles trapped at the outlet of the micro-pits may have rolled, which would have reduced the direct contact between the tool and the chips.

Black wear scratches were seldom found on the surfaces of the chips machined by the textured tools. Only in the case of the largest distance *d*_1_, in Figure 10k–o, did these wear traces appear; however they were much slighter than when machining with the non-textured tool (Figure 9). Furthermore, the scratch size was smaller. This phenomenon reflected the three-body friction during the machining. The micro-pitting-texture reduced the direct contact of the SiC particles with the rake face, thereby reducing the wear of the tool surface.

### 3.3. Cutting Force

**Figure 11:**
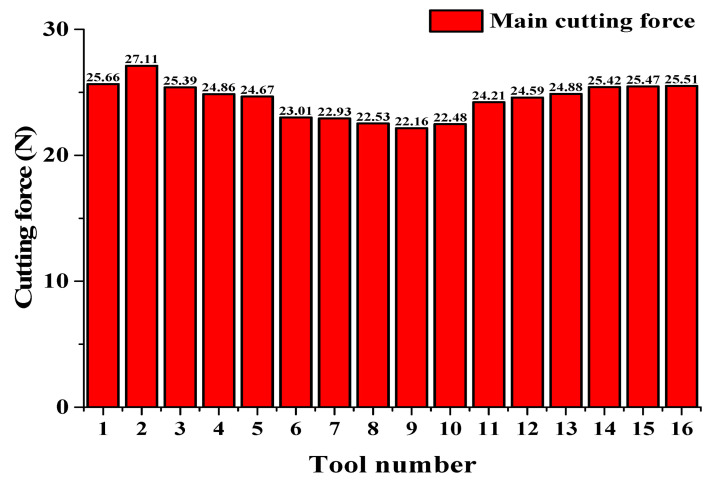
Main cutting force of non-textured Tool 1 and textured PCD Tools 2 to 16.

The cutting force was measured by a three-axis dynamic piezoelectric force-measuring instrument (Kistler 9256C, Winterthur, Switzerland) with a frequency 2 kHz. The main cutting forces of the non-textured tool and all the textured tools are shown in Figure 11. It can be seen that not all of the textures reduced the cutting force. When the micro-pitting-texture was too close to or too far from the main cutting edge, the cutting force was even greater than that of a non-textured tool (Tool 1 with *d*_1_ = 25 μm for *d*_2_ = 20 μm and Tool 16 with *d*_1_ = 45 μm for *d*_2_ = 100 μm). The reason for the first case probably lies in the increasing surface stress of the tool texture. In the latter case, the texture did not play an important role, since the texture was far from the intense interaction region, and the cutting force was approximately the same as that of a non-textured tool. The cutting force also did not change monotonically with pit spacing. The smallest cutting force appeared when the texture position and pit density were appropriate, with distances *d*_1_ = 35 μm and *d*_2_ = 60 μm (Tool 9 with cutting force reduction of 14% compared with the non-textured tool).

In micro-cutting, the texture parameters can change the contact state of the sticking and the sliding area. In the sliding area, the contact force and tool wear are lower because the friction coefficient is smaller in this region and the SiC particles roll in the interface. The sticking-area state mainly affects the chip-flow rate and the stress distribution at the tool–chip interface. The effect of the texture is reduced when the location of the micro-pits exceeds the sticking area, and it fails when the pits exceed the sliding area. The sizes of the sticking and sliding areas are the main factors affecting the cutting force. In the previous section (based on observations of the SEM images of the tool surface in Figure 8), it was found that the boundary between the sticking and sliding areas was at 35 μm to 45 μm from the main cutting edge. This is consistent with the fact that the smallest cutting force was that of Tool 9, with *d*_1_ = 35 μm. In addition, the error bars (standard deviation of cutting force) in Figure 11 indicate that the cutting stability of the main cutting force of the micro-pit-textured tool was quite good, in particular that of Tool 9.

### 3.4. Turning Force

The optimal texture obtained above with the smallest cutting force and low tool wear and adhesion (*d*_1_ = 35 μm and *d*_2_ = 60 μm) was then prepared on the surface of the turning tool (see pit distribution in Figure 3b). The experimental turning of this textured tool and of a non-textured tool on SiCp/Al cylinder bar was carried out.

**Figure 12:**
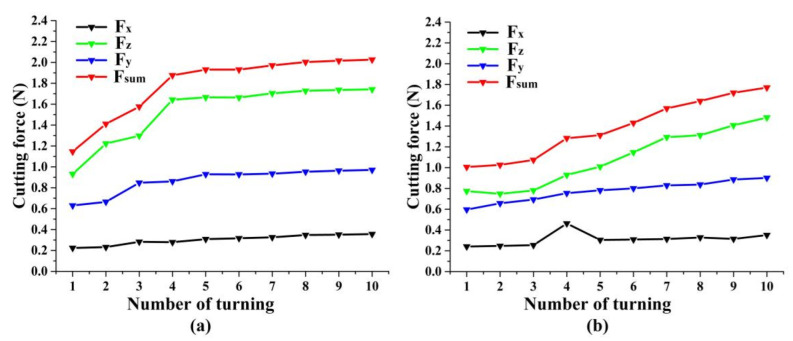
Turning forces of non-textured (**a**) and textured PCD tool (**b**) in 10 turnings.

Figure 12 shows the values of the cutting forces of the non-textured (Figure 12a) and textured tool (Figure 12b) in 10 turnings: *F*_z_ is the main cutting force in the direction perpendicular to the tool surface (green), *F*_y_ is the force parallel to the tool surface (blue), and *F*_x_ is the force in the feed direction (black). *F*_sum_ is the square root of sum of the squares of the cutting forces *F*_x_, *F*_y_, and *F*_z_ in all three directions (red). It can be seen that *F*_x_ is almost the same in two cases, and F_y_ is slightly lower in the case of the textured tool. The pits with rounded corners increased the flowability of the chip. The texture mainly affected the main cutting force F_z_. It was obviously reduced with the textured tool. The main reason for this is that the SiC particles played a rolling or supporting role between the tool and the chip, thereby reducing the cutting force.

**Figure 13:**
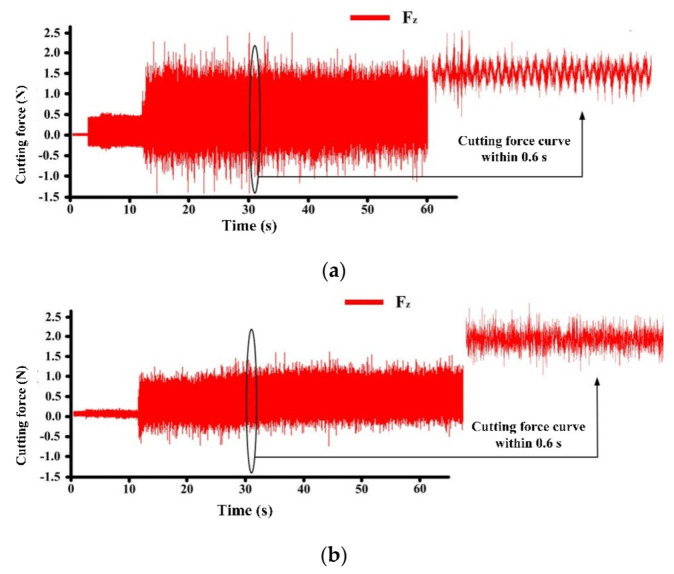
Main cutting force of non-textured PCD tool (**a**) and textured PCD tool (**b**) in turning experiment.

The main cutting forces *F*_z_ in the z-direction during one machining are shown in Figure 13. The reduction is clearly observable. The extracted data within 0.6 s also shows that the textured tool had a better cutting stability. The average *F*_sum_ value of the non-textured tool over 10 cuttings was 1.7887 N, while for the textured tool, it was 1.3831 N (reduced by 22%).

### 3.5. Chip State during Turning and Tool Surface after Turning

**Figure 14:**
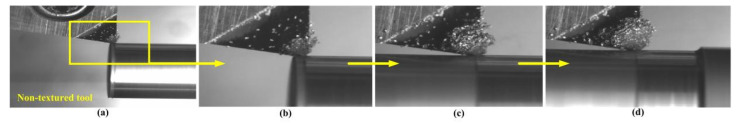
Snapshots of turning by non-textured tool. (**a**–**d**) show the chips in different turning time.

**Figure 15:**
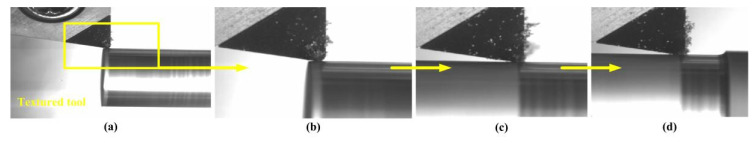
Snapshots of turning by micro-pit-textured tool. (**a**–**d**) show the chips in different turning time.

Snapshots taken at some stages of the turning process with the non-textured and textured tools are shown in Figure 14 and Figure 15. During the machining by the unstructured tool, the chips and the tool were always in an adhesive state (Figure 14b–d). Increasing numbers of chips collected at the tool tip. The adhesive wear of the tool was caused by the adhesion of the aluminum. The textured tool showed good chip evacuation and transport during the machining (Figure 15b–d). This resulted in reduced cutting force and longer tool life.

**Figure 16:**
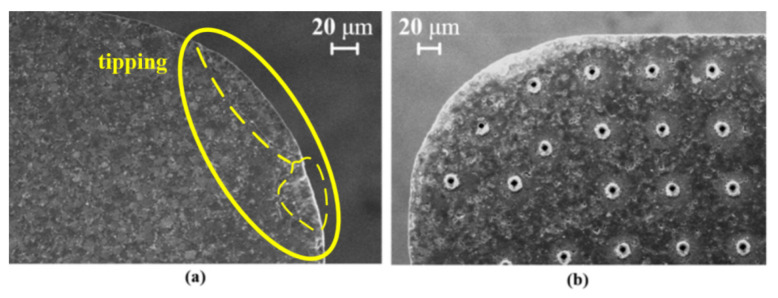
The SEM images of (**a**) non-textured PCD tool and (**b**) textured tool after 10 turnings.

After 10 turnings, tool tipping was clearly observed on the tip of the non-textured tool (Figure 16a), but it did not occur on the textured tool (Figure 16b). The micro-pits appeared clean and there were no clogs in the texture. Similarly to the orthogonal cutting experiment, the SiC third-body particles may have played a supporting and rolling role at the tool and chip surfaces, increasing the smoothness of the chip flow and reducing tool–chip contact and the possibility of tool tipping.

## 4. Conclusions

In this work, the cutting performance of textured and non-textured PCD tools on SiCp/Al composites was experimentally investigated. The micro-pits with rounded corners were prepared on PCD cutting and turning tools. The influence of the texture location and pit density on the cutting performance was studied. The tool wear, adhesion, and cutting force were evaluated. The conclusions are as follows:

1. The micro-pit array texture with rounded corners was formed by exploding the plasma on the surface of the PCD tool. These corners were designed to smooth the chip flow during the machining.
2. In the orthogonal cutting experiment, the textured PCD tool reduced the cutting force by 14% (Tool 9), but with an appropriate texture position (*d*_1_ = 35 µm) and pit density (*d*_2_ = 60 µm). This effect was not achieved if the texture was too close to or too far from the main cutting edge. The optimal texture was applied in the turning experiment; the cutting force was reduced by 22%, and no tipping or serious adhesion were observed.
3. The textured tool increased the chip curvature and reduced the black wear scratching of the SiC particles on the chip surfaces.
4. The tool wear, adhesion, and tipping were reduced with the textured tools. The main reason for this is probably the three-body interaction between the SiC particles, the chip, and the special-shaped texture.

## Figures and Tables

**Table 1:** Tool number and corresponding texture parameters.

Number	*d*_1_ (μm)	*d*_2_ (μm)	Number	*d*_1_ (μm)	*d*_2_ (μm)	Number	*d*_1_ (μm)	*d*_2_ (μm)
2	25	20	7	35	20	12	45	20
3	25	40	8	35	40	13	45	40
4	25	60	9	35	60	14	45	60
5	25	80	10	35	80	15	45	80
6	25	100	11	35	100	16	45	100
1	Non-textured tool							

**Table 2:** SiCp/Al material properties.

Density (g/cm^3^)	Elasticity Modulus (MPa)	Poisson’s Ratio	SiC Volume Fraction
2.89	158	0.25	45%
